# Prioritizing US Geological Survey science on salinization and salinity in candidate and selected priority river basins

**DOI:** 10.1007/s10661-024-13264-z

**Published:** 2024-12-16

**Authors:** Christopher H. Conaway, Nancy T. Baker, Craig J. Brown, Christopher T. Green, Douglas B. Kent

**Affiliations:** 1 US Geological Survey, Water Resources Mission Area, Moffett Field, CA USA; 2 US Geological Survey, Ohio-Kentucky-Indiana Water Science Center, Indianapolis, IN USA; 3 US Geological Survey, New England Water Science Center, East Hartford, CT USA

**Keywords:** Research design, Federal research, Hydrology, Basin selection, Salinity, Salinization

## Abstract

The US Geological Survey (USGS) is selecting and prioritizing basins, known as Integrated Water Science basins, for monitoring and intensive study. Previous efforts to aid in this selection process include a scientifically defensible and quantitative assessment of basins facing human-caused water resource challenges (Van Metre et al. in *Environmental Monitoring and Assessment*, *192*(7), 458 2020). In the present work, we explore this ranking process based on water quality considerations, specifically salinity and salinization. We selected top candidate basins to study salinity and salinization issues in 18 hydrologic regions that include 163 candidate basins. Our prioritization is based on quantitative assessment of sources of salinity, drivers of change, and receptors that must respond to those sources and drivers. Source terms represented in the prioritization include geology, depth to brackish groundwater, stream conductivity, chloride in precipitation, urban and agricultural land use, application of road salt as a deicer, and irrigation. Drivers represented in prioritization include changes in chemical weathering as a result of changes in rainwater chemistry. Receptors include measures of water stress, measurements of stream ecological health, and socioeconomic factors. In addition, we present research activities for the USGS on salinity and salinization that can be pursued in these basins including assessment of sources, pathways, and loadings; predicting and understanding changes in sources, peaks, and trends; understanding the components of salinity and mobilization of contaminants; understanding the relationship between salinization and changing ecosystems; and developing knowledge on the causes and distribution of groundwater salinity, brackish water resources, and challenges related to desalination.

**Supplementary Information:**

The online version contains supplementary material available at 10.1007/s10661-024-13264-z.

## Introduction

### Overview of salinity

One of the greatest challenges to the US in the twenty-first century is water availability. Increasing population and associated water use combined with warming temperatures and other anthropogenic activities across the US are driving greater aridity, particularly in western areas (Brown et al., 2019; Overpeck & Udall, 2020). At the same time, there is increasing freshwater salinization across the country (Kaushal et al., 2018). Salinity is the sum of dissolved salts in water, and whereas seawater has high concentrations of predominantly sodium and chloride ions, saline groundwater, brackish groundwater, and freshwater can have salinity from many other ions in various concentrations—for example, calcium, bicarbonate, magnesium, potassium, and sulfate (Kharaka & Hanor, 2014; Vengosh, 2014). Salinization can be a result of increases in any of these. The calculation of salinity and dissolved solids by summing the constituent concentrations using a geochemical modeling speciation code is considered the most comprehensive and accurate method because it accounts for the chemical speciation of all constituents (McCleskey et al., 2023). Increasing salinity of freshwater resources in the US poses a direct threat to ecological health and beneficial uses of water on a national scale (Cunillera-Montcusí et al., 2022; Dugan et al., 2017; Kaushal et al., 2021; Stets et al., 2020). Salinity and salinization as issues can broadly be broken into salinization of arid and semi-arid regions; salinization from agricultural practices such as irrigation and fertilizer application; salinization by application of road deicing products; salinization from sea-level rise and extensive pumping in coastal areas; aquifer weathering; and salinization coming from urban centers and urbanization through wastewater, runoff, and other sources (Anning & Flynn, 2014; Vengosh, 2014). Groundwater resources are often limited by salinity, and thus, understanding the geochemistry of brackish groundwater is essential in water security (Stanton et al., 2017). Increasing salinity can result in release of many toxic elements from aquifers or stream sediment, including geogenic contaminants such as radium (Kaushal et al., 2022; McMahon et al., 2019; Stackelberg et al., 2018; Szabo et al., 2012). Consequently, scientists and environmental managers must improve understanding of how and where to address issues of water availability and water quality as related to salinization.

### US national research priorities

Identifying national research priorities is of key importance to the US Geological Survey (USGS). Efforts to identify future and guide current research opportunities for water science in the USGS come from both within the agency (Evenson et al., 2013; Van Metre et al., 2020) and from expert external reviews, such as by the National Academy of Sciences (National Research Council 2009, 2012, 2018). Beginning in the 1990s, there was a multidecadal effort by the USGS to address water quality in the US called the National Water-Quality Assessment (NAWQA) Project. This project included an effort to understand water quality across the US in 51 US river basins and aquifers called “Study Units.” Guidance from the National Academy of Sciences was sought in developing recommendations for the third decade of NAWQA studies (National Research Council, 2012). In 2010, the Director of the USGS focused science activities of the organization into several mission areas, including the Water Mission Area (WMA), which most intersects with the issues of salinization and water resources. In 2013, the USGS WMA released a strategy for the following decade that included specific goals, objectives, and actions that were synthesized and prioritized into key areas (Evenson et al., 2013). In 2018, the National Academy of Sciences produced a review requested by USGS WMA to examine challenges over the following 25 years and determine research opportunities where the WMA could focus (National Research Council, 2018). In a complementary effort, the USGS WMA was developing a new programmatic approach combining intensive monitoring and assessment in selected hydrologic basins (Eberts et al., 2019), referred to by USGS as Integrated Water Science (IWS) Basins. A systematic, scientifically defensible approach for selecting these basins was developed by Van Metre et al. (2020). Our present work seeks to integrate these priority actions and basin selection methods for salinity and salinization specifically.

### Background and overview of research trends of salinity

Important reviews of the state of the science and gap analysis for salinity and salinization provide additional important background for our work (Cunillera-Montcusí et al., 2022; Kaushal et al., 2018, 2021; McMahon et al., 2016; Stanton et al., 2017; Vengosh, 2014). Vengosh (2014) presents a detailed review of salinization and saline environments covering rivers, lakes, and groundwater, with a special focus on natural sources versus the influence of anthropogenic processes. Kaushal et al. (2018) developed the notion of a freshwater salinization syndrome and the associated issues of corrosion and contaminant mobilization particularly in streams and rivers. Kaushal et al., (2021, 2022) summarize previous observations of salinization of freshwater, including groundwater resources and urban systems, develop the notion of the importance of “chemical cocktails” mobilized by increasing salinity, identify key scientific gaps, and propose specific management strategies. Based on their study of multidecadal trends of water quality constituents in streams across the conterminous US, Stets et al. (2020) suggested that research is needed to understand how increases in salinity might be affecting aquatic ecology and ecosystem services. Evenson et al. (2013) identified geologic controls and effects of climate variability on salinity as a key water quality concern to be addressed with specific actions. Evenson et al. (2013) also highlighted the importance of questions regarding the potential benefits and limitations of the use of “unconventional” water resources including saline and brackish groundwater. In a similar vein, data gaps and limitations regarding brackish groundwater as a resource, as well as specific recommendations for future research, are presented by Stanton et al. (2017). Cunillera-Montcusí et al. (2022) present a gap analysis combined with a broad, multiscale, and interdisciplinary research agenda to address freshwater salinization issues.

### Purpose and scope of this work

One strategy to address the challenges of salinity on water resources involves identifying key issues to inform use of USGS resources to solve these issues. Because resources are limited, the USGS must not only prioritize the development of tools and talent but must also prioritize geographical study areas where the tools and talent are most needed. With efforts connected and coordinated with various other science-based US federal agencies, one role of the USGS is to perform the hydrologic monitoring, laboratory-based experiments, and hydrological and geochemical modeling required to create a framework to guide US regulatory agencies, states and municipalities, non-governmental organizations (NGOs), and other stakeholders in their decision-making processes (Evenson et al., 2013). This role is intended to ensure that regulations and environmental management practices are effective, including the protection of water resources and ecological health. Indeed, the current water quality guidelines may be insufficient to protect lake ecosystems from salinization (Hintz et al., 2022). The effort by Van Metre et al. (2020) to prioritize research basins for the USGS based on systems with anthropogenic water quality stressors did not consider specific water quality components such as salinity, temperature, geogenics, or contaminants of emerging concern, although nutrients and geogenics have since been addressed (Erickson et al., 2024; Tesoriero et al., 2024). In the present study, we adopt a similar approach in prioritization, but include sources, drivers, and receptors specific to salinity and salinization. In addition, we consider the results of previous gap analyses and suggested research avenues (Cunillera-Montcusí et al., 2022; Evenson et al., 2013; Kaushal et al., 2021; National Research Council, 2018; Stanton et al., 2017) using our prioritization results. Although reviews are available that seek to guide the greater research community on science regarding salinization (e.g., Cunillera-Montcusí et al., 2022; Kaushal et al., 2021; Yihdego & Panda, 2017), this work focuses principally on information for the USGS WMA to prioritize science in USGS IWS Basins and elsewhere.

## Approach

### Rationale

To guide our selection process, we created three categories for our data sets: “Sources,” “Drivers,” and “Receptors.” These categories represent major known sources of salinity, the primary drivers of changes in salinity or salinization, and receptors where we deemed the influence of changing salinity to be of the most importance or most vulnerable. Prioritizing river basins for salinity research is then a balance of these categories. The higher priority basins then are places with greater potential for salinization and less potential for successfully adapting to the associated water quality challenge.

Ideas for variables related to the Source category were guided by considering the well-known main sources of salinity in the environment: natural sources (such as evaporite rocks), atmospheric deposition, road salt applications, irrigation, agriculture, and urban sources. Many of these are well known from literature (Anning & Flynn, 2014; Kaushal et al., 2018; Nauman et al., 2019; Vengosh, 2014), whereas others have also been identified in recent studies, such as the importance of urban sources (Kaushal et al., 2020; Stets et al., 2020). Some of the major drivers of salinization are well known and in scientific reviews. These include climate change, aridification, and changes in the hydrologic cycle. Deciding what the main receptors are for salinization issues requires some judgment as to what is important. To this end, we adopted much of the conceptual framework of Van Metre et al. (2020) on major factors affecting water availability in relation to the ranking approach and variables. We felt the most salient issues would fall under water stress or anthropogenic stress, ecosystem health, and the ability of communities to find resources to meet challenges and deal with future changes.

### Classification of regions and basins

**Fig. 1:**
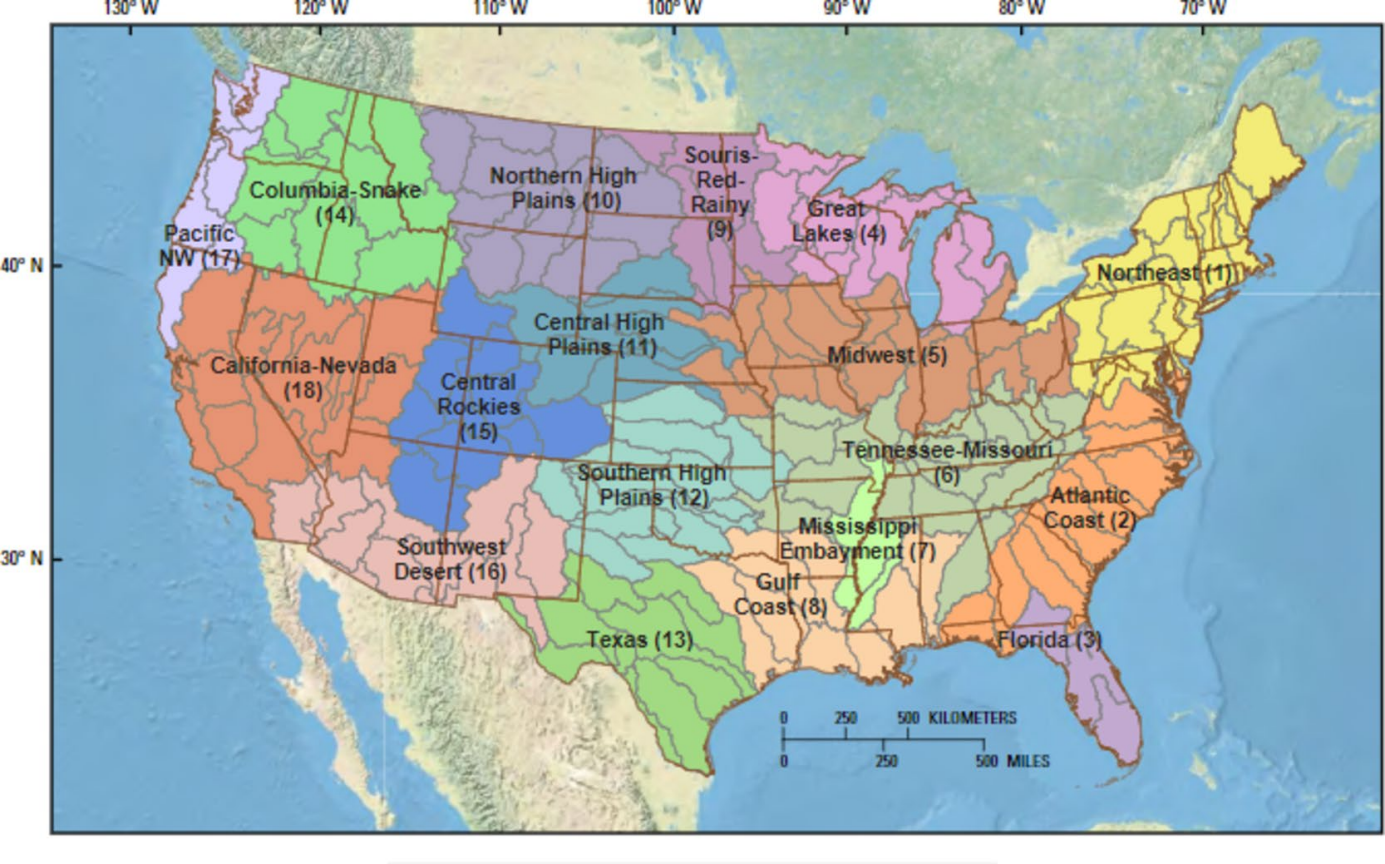
The 163 candidate basins and the 18 hydrologic regions (Van Metre et al., 2020) used for prioritizing study basins. The number in parentheses after the region name is assigned region number. The delineated areas within each hydrologic region are the individual basins. The borders of the 48 continental US states are also shown for reference. A more detailed map showing Basin ID numbers is presented as Supplementary Fig. 1

We followed the geographic classification used by Van Metre et al. (2020). This classification divides the US into regions—hydrologic landscape regions—based on large differences in climate and hydrology (Fig. 1). Basins or HUC4 watersheds are then assigned to those regions. As described in Van Metre et al. (2020), some smaller HUC4 basins were combined to provide 163 basins of approximately equal area. These modified HUC4 areas are hereafter referred to as “basins.” A list of basin names and Basin ID can be found in the Supplemental Tables, and a map showing each Basin ID is shown on Supplemental Figure S1.

### Data assembly

We reviewed the data selection process and also the data selected by Van Metre et al. (2020). Those data sets were reconsidered with respect to salinity. Major sources, drivers, and receptors for salinity were determined after review of current literature and based on the expertise of the team. The best data sets were identified based on availability, coverage, and workload required to attribute to the basins. Also considered were preliminary selection choices by other selection teams for geogenics (Erickson et. al, 2024), nutrients (Tesoriero et al., 2024), contaminants of emerging concern, and temperature. We preferred data sets that could be used across teams for other target constituents. For quality analysis and control, data sets were examined with histograms and correlation plots to identify data anomalies.

### Rationale for final data selection: Sources, Drivers, Receptors

The variables representing and falling within the categories of Sources, Drivers, and Receptors are described below and summarized in Table 1. The values for each of the variables below are presented in Table S1. A detailed description of data sources and how data were prepared and processed to the basin scale is provided in a USGS Data Release by Qi et al. (2023). All variables considered were aggregated from geospatial data by either averaging or summing to each HUC4 candidate basin.

**Table 1:** Datasets used in salinity rankings

Dataset (and category)	Short name	Description	Citation
Sources			
Karst in the US (combined evaporites and carbonate karst layers)	*Carbonate Evaporites*	Percent of the basin area underlain by soluble rocks with rock units containing significant amounts of carbonate or evaporite minerals	Weary & Doctor, 2014
Depth to brackish groundwater	*Depth to Brackish*	Average predicted depth to brackish groundwater in feet below land surface for each basin, processed to represent the percentage of area of the basin expected to have brackish water conditions between the surface and 1000 ft depth	Stanton et al., 2017
Predicted background conductivity	*Conductivity*	Stream length weighted mean predicted conductivity for each basin in μS/cm	Olson & Cormier, 2019
Chloride in wet deposition	*Rain Cl*	Three-year (2018–2020) average gradient map of precipitation-weighted mean wet deposition of Cl in rain samples in kg-Cl/ha	National Atmospheric Deposition Program (NADP), 2022a
National Land Cover 2019, CONUS: developed land	*Urban*	Percent of basin with Urban land use (classes include Developed, Open Space; Developed, Low Intensity; Developed, Medium Intensity; and Developed, High Intensity)	Dewitz and USGS, 2021
National Land Cover 2019, CONUS: agricultural land	*Ag*	Percent of basin with agricultural land use (classes include Pasture/Hay and Cultivated Crops)	Dewitz and USGS, 2021
Estimates of road salt application across the conterminous US, 1992–2015	*Road Salt*	Total sum of road salt applied from 1992 to 2015 in kg/km^2^	Bock et al., 2018
2015 Estimated Use of Water in the U.S.: Total freshwater withdrawals for Irrigation	*Irrigation*	Sum of total fresh surface water withdrawals for irrigation plus total fresh groundwater withdrawals for irrigation	Dieter et al., 2018
Drivers			
pH in wet deposition	*Rain pH*	Hydrogen ion concentration as pH from measurements made at the Central Analytical Laboratory, 2018	National Atmospheric Deposition Program (NADP), 2022b
Van Metre and others’ supplemental dataset	*Precipitation Change*	Mean precipitation percent change for 2070–2099 relative to the 1971–2000 mean based on RCP 8.5 emission scenarios, in mm	Van Metre et al., 2020
% Projected Change in Annual Evaporative Deficit	*Aridification*	Area weighted mean projected percent change in annual evaporative deficit in the basin	Environmental Protection Agency (EPA), 2022
Receptors			
Total freshwater withdrawals/runoff	*Water Stress*	Ratio of total freshwater use to runoff for each basin: water demand stress—two variables were included to calculate water demand stress: the ratio of Tot_WU to Runoff (*WU_Runoff* in Van Metre et al., (2020))	Dieter et al., 2018; Van Metre et al., 2020
Mean Aquatic Condition Score (2016)	*EcoHealth*	Mean probability that perennial stream reaches in the HUC12 would be rated as having “good” biological condition under the EPA National Rivers and Streams Assessment (NRSA). Lower values for this variable contribute to a higher overall ranking	Environmental Protection Agency (EPA), 2022
ESRI ArcGIS Living Atlas of the World; American Community Survey Median Household Income	*SocioEconomic*	Mean of median household income in the past 12 months (in 2020 inflation-adjusted dollars) by the US Census Block Group for each basin (table ID B19049). Lower values for this variable contribute to a higher overall ranking	ESRI ArcGIS Living Atlas of the World, 2022

#### Natural sources

We selected variables related to natural sources of salinity from geologic formations, groundwater, and atmospheric deposition. Because of the importance of evaporite deposits in providing Na, Ca, Cl, and SO4 and the importance of karst in carbonate contributions to natural salinity, we selected a variable (“*Carbonate Evaporites*”) representing combined evaporite and carbonate karst deposits in the US. The variable data are taken from Weary and Doctor (2014) and represent the percent of the basin areas underlain by rock units containing significant amounts of soluble carbonate or evaporite minerals. We selected a variable representing mean predicted natural conductivity in stream water (*“Conductivity”*) that uses the stream length weighted mean predicted conductivity for each basin. This variable is based on a model using several other predictors including geology (%CaO, %S), vegetation, and soils (Olson & Cormier, 2019). A variable representing depth to brackish groundwater (“*Depth to Brackish*”) was selected to represent issues related to groundwater availability and salinity challenges. The variable data are taken from Stanton et al. (2017) and represent the percentage of area of the basin expected to have brackish water conditions between the surface and 1000 ft depth. To capture the atmospheric sources of salinity, we selected a variable representing atmospheric deposition of chloride (*“Rain Cl”*). The data for this variable are from National Atmospheric Deposition Program (NADP) National Trends Network and represent the 3-year (2018–2020) average of precipitation-weighted mean wet deposition of Cl in each basin (National Atmospheric Deposition Program (NADP), 2022a). Trends in atmospheric deposition of salt ions have been noted as potentially important from a mass balance perspective in some regions such as the northeast (Kaushal et al., 2021). The pH of rainwater and thus atmospheric deposition of other acid anions are included in the Drivers variable category described below.

#### Anthropogenic sources

Variables selected included urban land use, agricultural land use, road salt application, and total freshwater withdrawals used for irrigation. An urban land use variable (“*Urban*”) uses the 2019 National Land Cover Database and represents the fraction of the basin area with *Urban* land use, including classes Developed, Open Space; Developed, Low Intensity; Developed, Medium Intensity; and Developed, High Intensity (Dewitz and Geological Survey, 2021). Here we note that our *Urban* variable is well correlated with many related metrics (data not shown), such as percent change of urban area over time, population, and percent impervious cover. Impervious cover, for example, could be considered for the Drivers category (Baker et al., 2019; Rossi et al., 2022), but we felt it was captured under the *Urban* variable. A variable for agricultural land use (“*Ag*”) was taken from the same dataset and represents the percent of each basin with agricultural land use that include classes Pasture/Hay and Cultivated Crops. The road salt application variable (“*Road Salt*”) represents an estimated total application by mass (kg) from 1992 to 2015 divided by basin area. The estimated loads have a spatial resolution of one-square kilometer across the conterminous US and are based on several data sources, which include road density and proportion of developed land use, depth and spatial extent of long-term snowfall, and the production and distribution of salt sources by state (Bock et al., 2018). We focused on total road salt application over 1992–2015 rather than an annual value in part to capture the potential legacy issues of road salt application (Cruz et al., 2022). For the irrigation data variable (“*Irrigation*”), we used the “2015 Estimated Use of Water in the U.S.: Total freshwater withdrawals for Irrigation” compiled by Dieter et al. (2018). Although irrigation could be considered a driver, we elected to include it as a source in our analysis as it likely reflects to some extent fertilizer applications and distribution.

#### Drivers

For our main Drivers data, we selected variables for rainwater pH, modeled changes in precipitation, and modeled aridification. Changes in atmospheric chemistry are connected to changes in chemical weathering of rocks (Kaushal et al., 2021; Xie et al., 2022); for example, Stets et al. (2014) found that increased alkalinity yields in large rivers were consistent with recovery from acidification. Neutralization of rainwater acidity by rocks can release cations that enter the aqueous environment, thus contributing to salinity (Driscoll et al., 2016). For the rainwater variable (“*Rain pH”*), we used data from the NADP for H ion concentration calculated from pH measurements of wet deposition in 2018 (National Atmospheric Deposition Program (NADP), 2022b). For our variable showing modeled changes in precipitation (*“Precipitation Change”*), we used the *PPT_Change* variable data from Van Metre et al. (2020). As described in that work, the variable is developed using mean estimates of 20 global climate models downscaled for the contiguous US, and change is determined as difference between years 2070 and 2099 relative to 1971–2000 based on the Representative Concentration Pathway 8.5 emissions scenario. We used an absolute value for %change, so that only magnitude of change mattered and not the direction of that change. We used a variable for aridification (*“Aridification”*) that represents % Projected Change in Annual Evaporative Deficit, which is similar to change in precipitation, but combines with temperature effects, and is specifically defined as the difference between potential evapotranspiration and actual evapotranspiration. The variable uses modeled average annual evaporative deficit during 2050–2074 relative to observed 1981–2010 conditions (Environmental Protection Agency (EPA), 2022). Salinization during drought or aridification can be caused by evapoconcentration, less dilution of saline water, changes in groundwater-surface water interaction, or movement of saline wedges in estuary with reduced freshwater flow (Mosley, 2015; Tweed et al., 2009; Vengosh, 2014). There is a mismatch in the time frame for predicted values of *Precipitation Change* and *Aridification*, but we do not expect this to confound our results. In total, these three Drivers are all related to predicted and measured large-spatial scale changes in hydrology and atmospheric chemistry that can act on sources of salinity.

#### Receptors

For variables in the Receptor category, we picked data representing stress on water availability, stream health, and socioeconomic factors. We selected a variable (“*Water Stress*”) related to the water stress variable (*WU_Runoff*) from Van Metre et al. (2020). Water stress is expected to add complexity to any sort of challenge from salinization. The variable represents the ratio of total freshwater use to runoff for each basin. The water use component data were taken from Dieter et al. (2018), and the runoff component data were taken from Van Metre et al. (2020). Basins with high *Water Stress* generally have local freshwater withdrawals similar to or exceeding locally generated runoff. High *Water Stress* values are found in arid settings with high water use demand such as the Lower Gila (Basin ID 1507) and Southern Mojave–Salton Sea (1810). Choosing an ecosystem variable proved challenging. The team wanted a common variable with good coverage across all basins and relevant to aquatic habitats. We considered a few data sets, including those used by Van Metre et al. (2020). We finally created a variable (“*EcoHealth*”) based on the Mean Aquatic Condition Score 2016 that predicts stream biological conditions under the EPA National Rivers and Streams Assessment (Environmental Protection Agency (EPA), 2022; Hill et al., 2017). The variable gives the area-weighted mean probability that perennial stream reaches in the HUC12 subbasins would be rated as having “good” biological condition. Therefore, lower values for this variable are given a higher ranking and thus contribute to a higher score. Similarly, choosing a socioeconomic variable proved challenging, and there was discussion given to the appropriateness of including socioeconomic drivers in our assessment and how to make a simple but not simplistic variable selection. We acknowledge and emphasize that there are many factors contributing to both individual and community-level ability to respond to water quality issues. Ultimately, the team selected a variable (*“SocioEconomic”*) using the US Census reported median annual income for the area. Source data for the variable are the mean within each basin of median household income in the past 12 months (in 2020 inflation-adjusted dollars) by the US Census Block Group (ESRI ArcGIS Living Atlas of the World, 2022). Our rationale for using these data is based on the presumption that areas with higher median income would have higher contribution to local government and thus better equipped to deal with changes in cost or infrastructure needs. Conversely, areas with low median income would face greater financial challenges in maintaining and replacing infrastructure or dealing with other water quality issues. Lower values for this variable are given a higher ranking and thus contribute to a higher overall score.

### Ranking process and data analysis

**Fig. 2:**
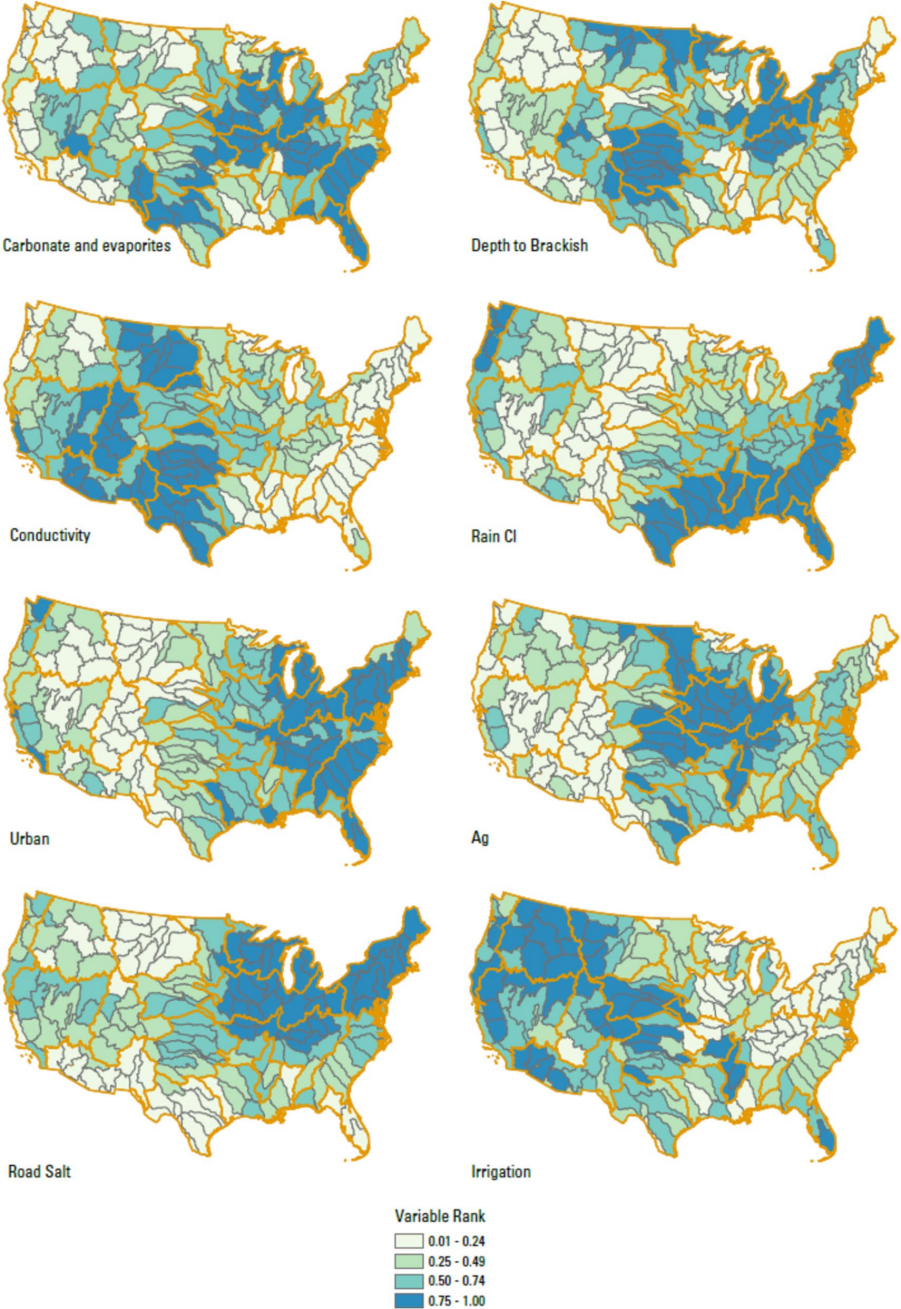
Map of the percent rank of values (scaled from 0 to 1) across the 163 basins for variables in the Sources category: *Carbonate and evaporites*, *Depth to Brackish*, *Conductivity*, *Rain Cl*, *Urban*, *Ag*, *Road Salt*, and *Irrigation*. Regional boundaries are highlighted in orange

**Fig. 3:**
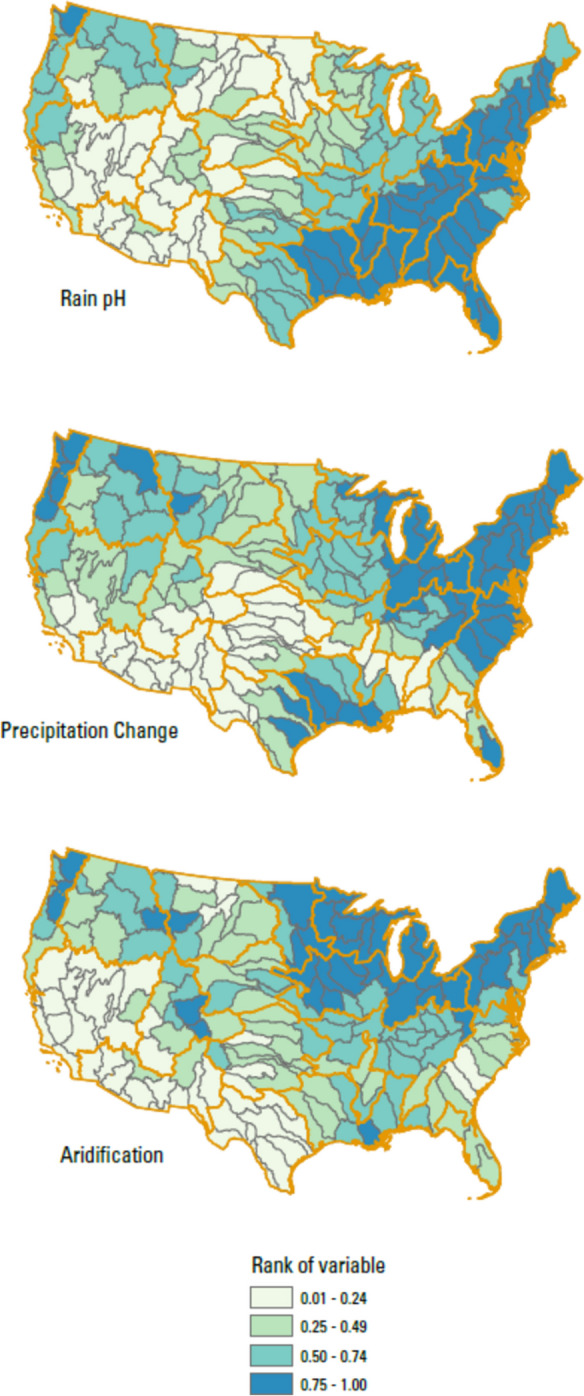
Map of the percent rank of values (scaled from 0 to 1) across the 163 basins for variables in the Drivers category: *Rain pH*, *Precipitation Change*, and *Aridification*. Regional boundaries are highlighted in orange

**Fig. 4:**
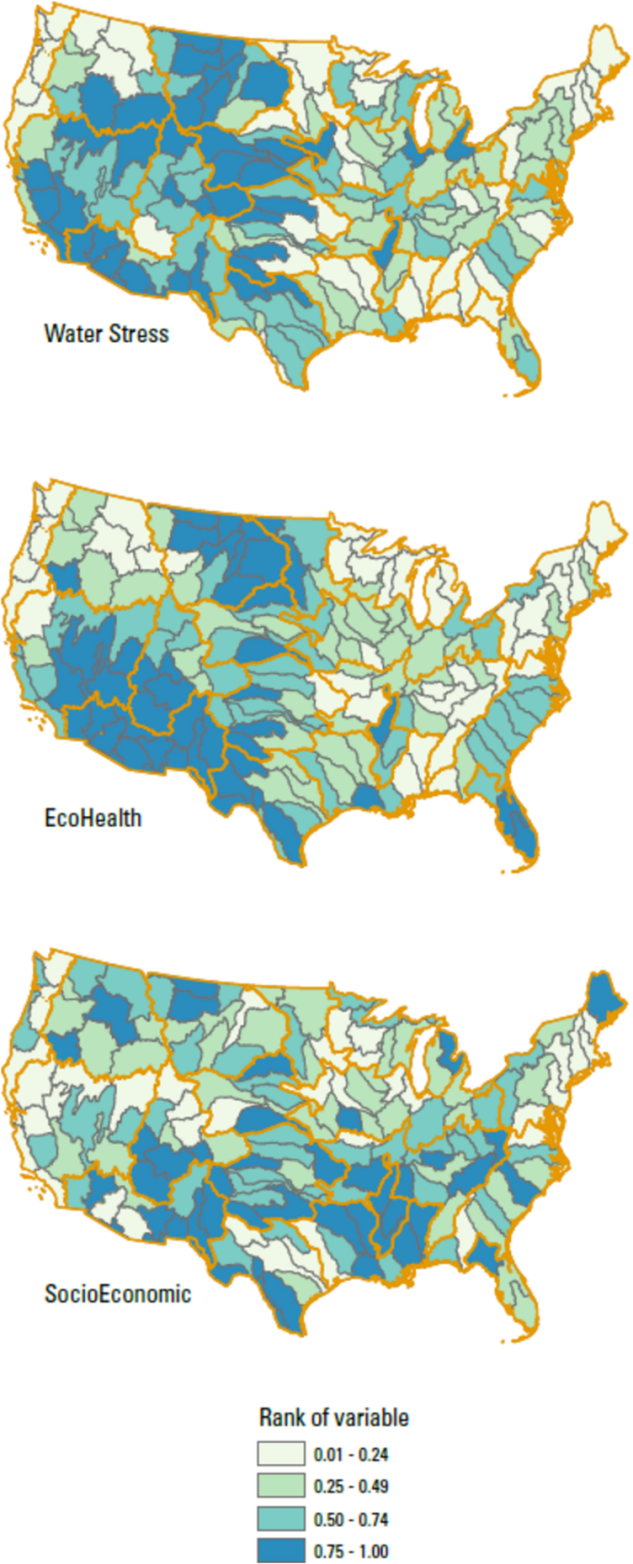
Map of the percent rank of values (scaled from 0 to 1) across the 163 basins for variables in the Receptors category: *Water Stress*, *EcoHealth*, and *SocioEconomic*. Regional boundaries are highlighted in orange

The calculations that we used with our assembled data followed the methods of Van Metre et al. (2020). For a given data set, we determined the percent rank excluding 0% and 100% of each value in the data set using the Microsoft Excel PERCENTRANK.EXC function. The percent rank was determined using values for all 163 basins. The percent ranks for each of the variables are shown on Figs. 2, 3, and 4. The given data set was then assigned a weighting to balance each of the main variable categories (i.e., Sources, Drivers, Receptors) evenly regardless of the number of variables within that category. For a given basin, all weighted values were summed to create a score for that basin. With each category ranked between 0.0 and 1.0, the possible range of the score, the sum of the three categories, was between 0.0 and 3.0. Each basin’s score was then ranked within its corresponding region, such that the top 2 basins could be identified within each region. We performed correlation tests between variables and scores by examining the Pearson correlation coefficient (*R*) as a preliminary screen for linear relationships with no test for outliers or data distribution. Statistical tests on correlations among data sets were conducted using Origin Pro 2018 or the Data Analysis Tools in Microsoft Excel (version 2202 in Microsoft 365 Apps for enterprise).

## Results

### Distribution of values for Sources, Drivers, and Receptors

The distribution of natural and anthropogenic Sources follows broad, regional patterns (Fig. 2). The *Carbonate Evaporites* variable reflects a combination of geological features explained by basin histories, such as the existence of ancient epicontinental seas. *Depth to Brackish* also reflects the distribution of geologic terrains, with high-ranking basins typically in the broad region between the Appalachian and Rocky Mountains. The distribution of *Conductivity* is generally related to a combination of marine sedimentary rocks and modern arid conditions. *Rain Cl* shows the influence of the ocean on atmospheric chemistry, with coastal areas being the highest ranked. The distribution of *Urban* values highlights the highly developed coastal regions and Midwest. *Ag* values show the primary use of land in the mid-continent as agricultural. *Road Salt* shows the highest use around the Great Lakes and in the Northeast, as well as a mid-latitude band across the US. *Irrigation* shows the importance of irrigation in the West and water diversions in the Florida region. There are many statistically significant correlations between Sources variables (Table 2) but few with a strong relationship (*R* > 0.75). A strong correlation (*R* = 0.80) exists between *Rain Cl* and *Rain pH*, which may reflect similar influences on atmospheric chemistry of rainwater.

**Table 2:** Pearson correlation coefficient (*R*) for the 14 percentile-ranked variables

	*Carbonate Evaporites*	*Depth to Brackish*	*Conductivity*	*Rain Cl*	*Urban*	*Ag*	*Road salt*	*Irrigation*	*Rain pH*	*Precipitation Change*	*Aridification*	*Water Stress*	*EcoHealth*	*SocioEconomic*
*Carbonate Evaporites*	1.00													
*Depth to Brackish*	0.11	1.00												
*Conductivity*	− 0.06	0.30*	1.00											
*Rain Cl*	0.14	− 0.34*	− 0.50*	1.00										
*Urban*	0.27*	− 0.04	− 0.50*	0.51*	1.00									
*Ag*	0.35*	0.34*	− 0.16*	− 0.10	0.19*	1.00								
*Road Salt*	0.17*	0.23*	− 0.33*	0.01	0.54*	0.25*	1.00							
*Irrigation*	− 0.22*	− 0.29*	0.06	− 0.11	− 0.23*	− 0.07	− 0.22*	1.00						
*Rain pH*	0.19*	− 0.23*	− 0.66*	0.80*	0.50*	− 0.03	0.18*	− 0.17*	1.00					
*Precipitation Change*	0.01	− 0.12	− 0.63*	0.48*	0.46*	0.04	0.52*	− 0.16*	0.49*	1.00				
*Aridification*	0.08	0.09	− 0.54*	0.04	0.20*	0.30*	0.51*	− 0.19*	0.19*	0.61*	1.00			
*Water Stress*	− 0.22*	− 0.06	0.35*	− 0.24*	− 0.19*	− 0.10	− 0.16*	0.30*	− 0.33*	− 0.37*	− 0.39*	1.00		
*EcoHealth*	0.06	− 0.12	− 0.64*	0.15	0.25*	0.07	0.30*	− 0.08	0.33*	0.60*	0.65*	− 0.45*	1.00	
*SocioEconomic*	− 0.07	− 0.08	− 0.16*	0.11	0.50*	0.03	0.20*	− 0.01	− 0.06	0.32*	0.16*	− 0.04	0.20*	1.00

Pearson correlation coefficient

^*^Significant at *p* < 0.05

Variables in the Drivers category show regional patterns (Fig. 3). The distribution of *Rain pH* values shows the influence of urban areas and the predominant wind direction to the East across the US. *Precipitation Change* shows that the changes of precipitation relative to current values are predicted to occur in the Northeast and Pacific Northwest. The distribution of *Aridification* values is somewhat like *Precipitation Change*, and there is a moderate (*R* = 0.61) significant correlation between the two variables. It is important to consider that *Aridification* is based on relative percent change rather than absolute changes. Whereas changes in arid regions might be greater on an absolute scale (change in precipitation depth per unit time), the relative changes are what are shown here (change in precipitation depth per unit time divided by the historical precipitation depth value).

Variables in the Receptors category show a somewhat more patchwork distribution of values (Fig. 4), although still regionally influenced. *Water Stress* appears to somewhat reflect areas that combine irrigation and low precipitation. *EcoHealth* shows a broad distribution of low values in the western US and a patchwork of high and low values in the eastern US. This may reflect the additional stress of higher aridity and conductivity on stream health in the West. *EcoHealth* has moderate (0.5 < *R* < 0.75) significant correlations with *Conductivity* (*R* =  − 0.64) and *Aridification* (*R* = 0.65). The *SocioEconomic* variable distribution shows higher mean median income areas of the West Coast and Northeast. Lower mean median income areas appear to be somewhat distributed across Appalachia and the Sun Belt regions of the US.

### Ranking results

**Fig. 5:**
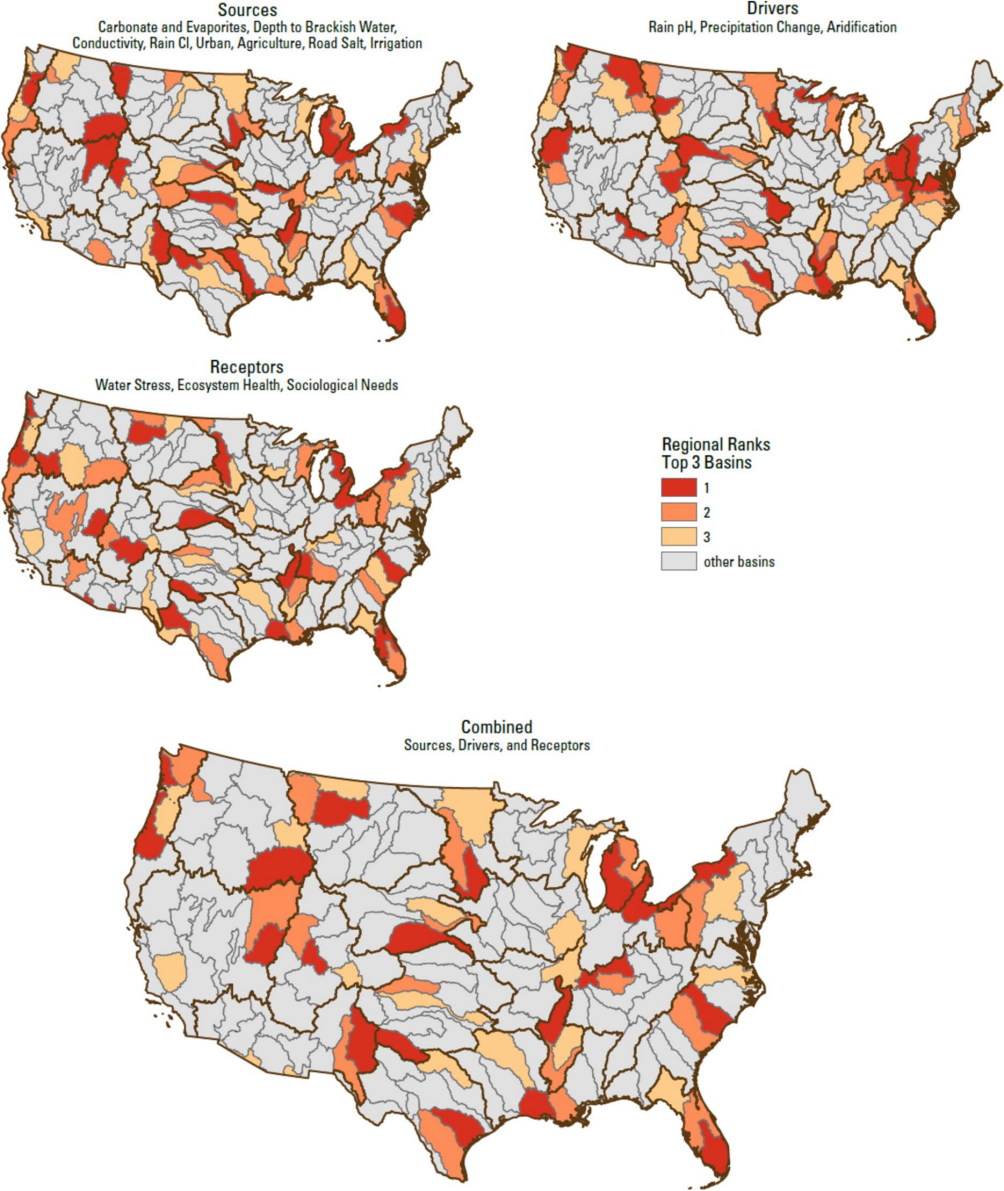
Map of top three basins within each of the 18 regions. Smaller maps show the top three basins based on subscores for Sources, Drivers, and Receptors. The large map shows the top 3 basins based on the combined final score for all categories. Regional boundaries are shown in black

**Fig. 6:**
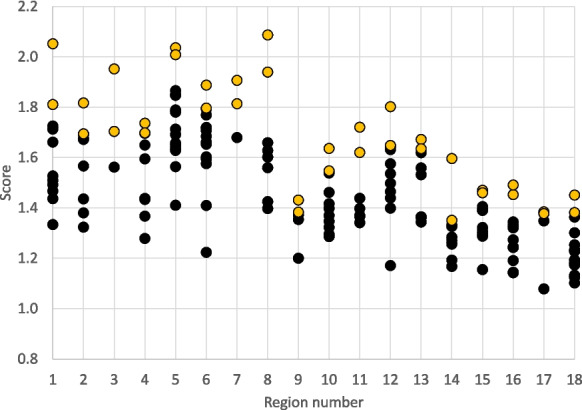
Distribution of final score values within the 18 regions. The top two basin scores within each region are highlighted in orange

The numerical rankings for the 163 basins provide a scientifically informed approach for identifying basins where salinity research could be prioritized. A complete list of variables and their values are given on Supplementary Table S1, and the percent rank of each of these variable values is given on Supplementary Table S2, and a plot of the top 3 basins in each region is shown on Fig. 5. Correlation between the final selected data was examined using Pearson’s correlation matrix (Table 2). The top two ranked basins for each region are given below on Table 3, with a complete list of scores and rankings on Supplementary Table S3. The value of scores ranged from 1.08 to 2.09. The numerical distribution of scores within each basin is shown on Fig. 6, highlighting how close the scores are compared to the ranked values. To understand what categories and variables were most important in influencing final scores within each region, we examined the Pearson product-moment correlation coefficient (*R*) between the percent rank for each variable and the final numerical score (Table 4). Similarly, we examined the correlation coefficient between category subscores—the sum of variables under each category for Sources, Drivers, and Receptors—and the final numerical score. Comparisons are also presented with the ranking results from Van Metre et al., (2020, 2023).

**Table 3:** Top-two candidate basins in each hydrologic region based on numerical ranking. The location of each basin by Basin ID is given on Supplemental Fig. 1

Region name (number)	Basin ID	Basin name	Rank
Northeast (1)	411	Lake Erie and Ontario	1
	501	Allegheny-Monongahela	2
Atlantic Coast (2)	304	Pee Dee	1
	305	Edisto-Santee	2
Florida (3)	309	Southern Florida	1
	308	Florida northcentral	2
Great Lakes (4)	405	Eastern Lake Michigan	1
	407	Lake Huron	2
Midwest (5)	409	Western Lake Erie	1
	503	Upper Ohio	2
Tennessee-Missouri (6)	514	Lower Ohio	1
	511	Green	2
Mississippi Embayment (7)	802	Lower Mississippi-St. Francis	1
	805	Boeuf-Tensas-Big Black	2
Gulf Coast (8)	808	Louisiana Coastal	1
	807	Lower Mississippi	2
Souris-Red-Rainy (9)	1017	Missouri-Big Sioux	1
	1016	James	2
Northern High Plains (10)	1004	Missouri-Musselshell	1
	1003	Missouri-Marias	2
Central High Plains (11)	1025	Republican	1
	1020	Platte	2
Southern High Plains (12)	1205	Brazos Headwaters	1
	1104	Upper Cimarron	2
Texas (13)	1210	Central Texas Coastal	1
	1211	Nueces-Southwestern Texas Coastal	2
Columbia-Snake (14)	1704	Upper Snake	1
	1703	Yakima	2
Central Rockies (15)	1403	Upper Colorado-Dolores	1
	1406	Lower Green	2
Southwest Desert (16)	1306	Upper Pecos	1
	1305	Rio Grande Closed Basins	2
Pacific Northwest (17)	1710	Oregon-Washington Coastal	1
	1711	Puget Sound	2
California-Nevada (18)	1603	Escalante Desert-Sevier Lake	1
	1601	Great Salt Lake	2

**Table 4:** The Pearson correlation coefficient (*R*) among basins for percent rank of variables and subscores versus final score

		Source								Driver			Receptor			Category subscores		
		*Carbonate Evaporites*	*Depth to Brackish*	*Conductivity*	*Rain Cl*	*Urban*	*Ag*	*Road Salt*	*Irrigation*	*Rain pH*	*Precipitation Change*	*Aridification*	*Water Stress*	*EcoHealth*	*SocioEconomic*	Source	Driver	Receptor
Region number	Region name																	
1	Northeast	0.33	0.82*	0.89*	− 0.45	0.32	0.77*	0.70*	0.14	0.02	− 0.73*	0.01	0.62*	0.84*	0.37	0.81*	− 0.14	0.93*
2	Atlantic Coast	− 0.12	0.38	0.68*	0.04	0.15	0.34	0.15	− 0.41	− 0.73*	0.89*	− 0.36	0.66	0.42	0.51	0.28	0.66	0.86*
3	Florida	− 0.36	1.00*	0.97	0.96	0.70	1.00*	− 0.81	1.00*	− 0.92	1.00*	− 0.49	0.96	0.64	− 0.94	1.00*	0.99	0.25
4	Great Lakes	0.90*	− 0.13	0.45	0.74*	0.86*	0.68*	0.82*	0.61	0.63	0.37	− 0.72*	0.59	0.34	− 0.04	0.91*	0.15	0.76*
5	Midwest	− 0.19	0.54*	− 0.41	0.51	0.65*	− 0.58*	0.67*	0.04	0.67*	0.56*	− 0.26	0.54*	0.44	0.25	0.54*	0.55*	0.68*
6	Tennessee-Missouri	0.23	0.40	0.35	− 0.59*	0.15	0.36	0.89*	− 0.35	− 0.24	0.74*	0.70*	0.48	0.00	0.26	0.57*	0.67*	0.51
7	Mississippi Embayment	0.34	0.88	0.98	− 0.29	0.41	0.60	1.00*	0.27	− 0.83	0.06	− 0.24	0.72	0.59	− 0.58	0.76	− 0.16	0.70
8	Gulf Coast	− 0.70	0.28	0.13	0.47	− 0.02	0.30	0.52	0.39	0.31	0.66	0.57	0.69	0.93*	− 0.03	0.21	0.78*	0.91*
9	Souris-Red-Rainy	0.92*	− 0.48	0.31	0.64	0.20	0.76	0.67	0.87	0.50	0.26	0.35	0.68	− 0.58	0.02	0.89*	0.39	− 0.14
10	Northern High Plains	0.26	− 0.01	− 0.41	− 0.31	− 0.38	0.13	− 0.48	0.57	0.58	0.22	0.01	0.15	− 0.21	0.90*	0.26	0.31	0.43
11	Central High Plains	0.81*	0.58	− 0.29	0.55	0.25	0.81*	0.69	0.35	0.16	− 0.64	0.35	0.42	0.35	0.17	0.79*	− 0.20	0.73
12	Southern High Plains	− 0.25	0.02	0.24	− 0.24	0.03	0.33	0.06	0.70*	− 0.24	0.30	− 0.53	0.83*	0.77*	0.37	0.25	− 0.34	0.86*
13	Texas	− 0.29	− 0.22	− 0.59	0.54	0.72*	0.78*	0.16	0.17	0.56	0.57	0.60	0.59	− 0.29	− 0.22	0.59	0.60	0.02
14	Columbia-Snake	0.50	0.13	0.60	0.27	0.05	0.24	0.11	0.10	− 0.17	0.22	0.35	0.48	0.20	0.02	0.50	0.17	0.36
15	Central Rockies	− 0.48	0.06	− 0.23	0.20	0.17	0.43	0.46	0.64*	0.37	0.15	0.50	0.67*	− 0.45	− 0.07	0.28	0.45	0.04
16	Southwest Desert	0.57	0.82*	0.62*	− 0.41	− 0.49	− 0.25	0.14	− 0.36	− 0.17	0.61*	0.02	0.09	0.73*	0.67*	0.38	0.06	0.65*
17	Pacific Northwest	− 0.95	0.40	− 0.79	0.99*	0.62	0.17	− 0.70	− 0.53	0.76	0.90	0.84	− 0.59	0.77	0.11	− 0.46	0.87	0.13
18	California-Nevada	− 0.06	− 0.20	0.04	0.17	− 0.06	0.50	0.52	0.58*	− 0.12	0.20	0.64*	0.46	− 0.13	0.43	0.46	0.20	0.36

^*^Significant at *p* < 0.05

#### Northeast Region (1)

Increased salinization of surface water is a known major issue in the northeastern US and is predominantly associated with road salt, urbanization, and increasing impervious surface coverage (Anning & Flynn, 2014; Kaushal et al., 2005, 2018; Rossi et al., 2022, 2023). The *Road Salt* percent ranks for basins in this region, with a median value of 0.93, reflect the importance of this source (Table S2). The top scoring basin in this region is the Lake Erie and Ontario Basin (Fig. 5), which includes the southern shore of Lake Erie and Lake Ontario. The top score is an outlier (Fig. 6), and that score is driven in large part by a low Mean Aquatic Condition Score, which translates to a relatively high *EcoHealth* rank (Fig. 3, Supplementary Tables S1, S2). This area of low predicted probabilities of good biological condition is also illustrated in Fig. 5 of Hill et al. (2017). Underlying variables of importance to the Mean Aquatic Condition Score in the region, described under the Northern Appalachians in Appendix S3 of Hill et al. (2017), are metrics of water impoundment such as dam density and dam volume and human population density. The second ranked basin for salinity in the region is the Allegheny-Monongahela, which is predominantly in western Pennsylvania, and also has a standout score (Fig. 6) that is driven by the variables in the Drivers category (Fig. 5, Tables S2, S3), particularly *Aridification*.

#### Atlantic Coast Region (2)

Relatively little road salt is used in the southeastern US; thus, salinization of freshwater streams is thought to be primarily related to changes in chemical weathering and other sources (Anning & Flynn, 2014; Kaushal et al., 2018). There are also salinity challenges in this region that are related to seawater intrusion threating groundwater resources (Jasechko et al., 2020). The top scoring basin in this region is the Pee Dee Basin (Fig. 5), which has its headwaters in North Carolina and drains to the Atlantic in South Carolina. The score is driven by values in the Receptor category (Fig. 4), especially *Water Stress* and *SocioEconomic* variables. The Pee Dee Basin has the lowest mean median income in the region (Table S1). The second ranked basin in the present study is the adjacent Edisto-Santee, although its score (1.694) is almost indistinguishable from the third ranked Chowan-Roanoke Basin (1.692), which straddles the North Carolina-Virginia border, and the second to fifth ranked basins have scores that are all relatively close to one another (Fig. 6). This similarity in scores mirrors the similarity in Sources and Drivers across much of the region.

#### Florida Region (3)

The Southern Florida Basin is top ranked in this region (Fig. 5). There are only three basins in this region, and the Southern Florida Basin stands out both in respect to variables in the Sources category and climate change related Drivers (Figs. 2 and 3). More specifically, high values for variables *Depth to Brackish*, *Conductivity*, *Irrigation*, and *Precipitation Change* make the Southern Florida Basin stand out (Table 4). Within the Receptor variables, a high *Water Stress* rank in the Southern Florida Basin is offset by a relatively high mean median income for the region. The Southern Florida Basin overlaps with the Southern Florida NAWQA study unit. Because of the shallow aquifers and porous limestone in this area, groundwater is vulnerable to surface contamination and to saltwater intrusion (McPherson et al., 2000). In addition, the role of salinity in distribution of invasive fishes with broad salinity tolerance was also noted.

#### Great Lakes Region (4)

Increased salinization of freshwater lakes and groundwater is a known major issue in the Great Lakes Region and is predominantly associated with road salt and water softener brines (Dugan et al., 2017; McDaris et al., 2022; Rayne et al., 2019). The Eastern Lake Michigan Basin is top ranked in this region. The differences in Sources category variables such as *Carbonate Evaporites*, *Urban*, and *Road Salt* appear to drive rankings within this region, with significant linear (Pearson) correlation coefficients between those variables and the total score being 0.90, 0.86, and 0.82, respectively (Table 4). Scores in the second- and third-ranked basins are practically indistinguishable (Lake Huron Basin 1.698 and Western Lake Michigan Basin 1.696), with scores decreasing steadily for the remaining basins (Fig. 6).

#### Midwest Region (5)

Salinity increases have been observed in the midwestern US lakes and streams and groundwater (Dugan et al., 2017; Kaushal et al., 2018), and the Midwest Region in this study is a nexus of relatively high values for major sources of salinity, *Ag*, *Road Salt*, and *Urban* (Fig. 2). The Western Lake Erie Basin has the top score in this region. That basin’s score stands out (Fig. 6) because of relatively high values for *Water Stress*, *EcoHealth*, and *Depth to Brackish*. It may be argued that brackish groundwater resources are not a significant issue in the Western Lake Erie Basin; however, excluding *Depth to Brackish* in the ranking exercise yields a similar ranking because of relative differences in *EcoHealth* and *SocioEconomic* variables. The Western Lake Erie Basin overlaps with a NAWQA Study Basin, Lake Erie-Lake Saint Clair Drainages (Myers et al., 2000). Highlights in the results of the NAWQA study in that area include the recognition that recent residential development in the Detroit area has had a widespread effect on groundwater salinity, likely from road salt, septic systems, and backwash from water softeners. Use of chloride/bromide ratios were used to distinguish natural sources from anthropogenic ones in the study unit (Davis et al., 1998). The Upper Ohio, which is predominantly in the eastern part of Ohio, ranks second, also has a standout score because of relatively high variable values in the Drivers category. The Upper Illinois Basin, the 4th ranked basin in this region, overlaps with the NAWQA study unit Upper Illinois River Basin, where a correlation between increasing chloride and urban land use and decreasing number of benthic invertebrate species sensitive to pollution was highlighted in previous work (Groschen et al., 2004).

#### Tennessee-Missouri Region (6)

The Lower Ohio Basin, which straddles the Indiana-Kentucky border, is top ranked in the region, with the Drivers category being an important part of the scores overall in the region (Table 4, *R* = 0.67), particularly due to *Precipitation Change* and *Aridification* (Table 4, *R* = 0.74 and 0.70, respectively). The relatively high *Water Stress* variable (Table S2, rank 0.59) also contributes to this basin’s high score. The top scoring basins in this region are all in the northern part of the region, with the second ranked Green Basin being just south of the Lower Ohio Basin. The Sources values in the northern part of the region are similar to the Midwest Region above (Fig. 2), and therefore, salinity issues may be similar. The Sources category shows a moderate correlation (*R* = 0.57) with overall score, with *Road Salt* being a strong contributor (*R* = 0.89).

#### Mississippi Embayment Region (7)

The Lower Mississippi-St. Francis Basin, which is mostly within northeast Arkansas, is top ranked in the region. There are only three basins in this region; however, differences in Sources category (Table 4, *R* = 0.76) and the *Water Stress* variable (Table S2, rank 0.87) appear to be determinative. The relatively high values for *Water Stress* variables coincide with increases in irrigation and heavy groundwater extraction in that area (Yasarer et al., 2020). The Lower Mississippi-St. Francis Basin is within the NAWQA Mississippi Embayment study unit. Groundwater quality in that study unit was characterized as generally very good, while highlighted issues in stream water quality include pesticides, phosphorus, and organochlorines (Kleiss et al., 2000).

#### Gulf Coast Region (8)

The Louisiana Coastal Basin, in southwest Louisiana, is top ranked in this region. The second-ranked Lower Mississippi Basin, which is just to the east of the Louisiana Coastal Basin, also has a relatively high score (Fig. 6). Overall scores in the region are strongly correlated with the Receptors (Table 4, *R* = 0.91) category and the *EcoHealth* variable (*R* = 0.93). The top 2 basins have relatively high scores for changes in modeled precipitation (*Precipitation Change*) compared to other basins in the region (Table S2). The Louisiana Coastal and Lower Mississippi basins overlap the western and eastern part of the NAWQA Acadian-Pontchartrain Drainages study unit. Highlighted water quality issues in that study unit included pesticides, herbicides, and phosphorus in small streams and rivers, radon in groundwater, and the existence of canals that are part of oil and gas infrastructure linked with saltwater intrusion into historically fresh marshes in the southeast of the study unit (Demcheck et al., 2004). Although not captured in our ranking study, in southern Arkansas and northeastern Louisiana, roughly corresponding with the Lower Red-Ouachita Basin, groundwater withdrawals for municipal and industrial use from the middle Claiborne aquifer have caused saltwater to move into the aquifer, causing concerns for long-term availability of freshwater in the region (Kingsbury et al., 2014).

#### Souris-Red-Rainy Region (9)

Salinity increases in surface water in this region have been documented and related to excess nitrogen and phosphorus from fertilizer application (Kaushal et al., 2018), which is expected to be reflected by the *Ag* variable (Fig. 2), and chloride increases attributed to road salt (Dugan et al., 2017). The Missouri-Big Sioux Basin is top ranked in this region. Although there are few basins in this region (*n* = 5) to perform statistical analysis, the Sources category appears to be an important contributor to overall score (Table 4, *R* = 0.89), where *Carbonate Evaporites* stand out as a source driver. The Missouri-Big Sioux Basin, which represents a predominantly agricultural region in eastern South Dakota, has a score that stands out in part because of relatively high value for *Water Stress* and *Irrigation* ranks (Table S2). Within the other basins in the region, there are comparable ranks in many respects (high road salt use, high irrigation, high agricultural land use), and the scores are similar (Fig. 6) among ranks 2, 3, and 4. The Souris Basin, located in north-central North Dakota, has the lowest score and is characterized by low *Carbonate Evaporites*, *Road Salt*, and *Irrigation* (Table S2).

#### Northern High Plains Region (10)

The Missouri-Musselshell, which is east of the Continental Divide in central Montana, is top ranked in the region, and it stands out because of its relatively low *SocioEconomic* variable value (Fig. 4) paired with high climate-related Drivers compared to other basins in the region. Scores in the second- and third-ranked basins are relatively close (Missouri-Marias and Milk Basins), with scores decreasing steadily for the remaining basins and the *SocioEconomic* variable appears to be an important contributor (Table 4, *R* = 0.90).

#### Central High Plains Region (11)

The Republican Basin, which covers much of the Nebraska-Kansas border and into Colorado, is top ranked in the region, driven by the consistently high variable values across the Receptor category (Table S2) giving a relatively high subscore in that subcategory (Table S3). Second-ranked Platte Basin, which is just to the north of the Republican Basin, also has a relatively high total score for the region (Fig. 6) that is driven by Sources including *Carbonate Evaporites*, *Depth to Brackish*, and *Ag* (Table S2). The overall score for basins in the region is significantly correlated with the Sources category (Table 4, *R* = 0.79). The Republican Basin and much of the Central High Plains Region overlap with the northern part of the NAWQA High Plains Regional Groundwater study unit (Gurdak et al., 2009). Results from that work show that in the northern part of the High Plains the groundwater is generally acceptable for drinking-water purposes with respect to dissolved solids, major ions, and trace elements (Stanton & Fahlquist, 2006). Dissolved solids concentrations in groundwater are influenced by irrigated agriculture in some of these areas, but natural sources such as water rock interaction predominate below the zone of agricultural influence (McMahon et al., 2007).

#### Southern High Plains Region (12)

The Brazos Headwaters Basin, which runs through Texas and is at the southern edge of the High Plains regions, is top ranked out of the 11 basins of the region. The differences in scores for the eleven basins in this region are best correlated with *Water Stress* and *EcoHealth* variables (Table 4). The score for the Brazos Headwaters Basin stands out (Fig. 6) because of relatively high *Water Stress* and *EcoHealth* rank values (Table S2). Thus, the selection is guided by the basin being the most vulnerable to disturbance in the region. The second- to fourth-ranked basins have similar scores to one another, with scores for basins decreasing steadily in the remaining basins. The Central High Plains Aquifer, which lies within the Southern High Plains Region, has areas in contact with underlying geologic formations that contain deep saline groundwater (Nativ & Smith, 1987), and natural upwelling here can cause salinization (DeSimone et al., 2014). To the south, the Brazos Headwaters Basin and much of the Southern High Plains Region overlap with some of the southern part of the High Plains Aquifer (Gurdak et al., 2009), where dissolved solids concentrations, as well as sulfate and chloride, are highlighted as water quality concerns in this part of the study unit (Stanton & Fahlquist, 2006), and processes such as evaporative concentration and conversion of rangeland to irrigated cropland are thought to influence groundwater quality through changes in weathering of natural subsoil salt deposits and irrigation return flow (McMahon et al., 2007).

#### Texas Region (13)

The Central Texas Coastal Basin, which includes the northern part of the Texas Gulf Coast, is top ranked out of the 9 basins of this region. The scores in this region are not significantly related to any of the category subscores (Table 4) but are significantly correlated with *Urban* and *Ag* variables. The Central Texas Coastal Basin and the Nueces-Southwestern Texas Coastal Basin to the south (which ranks second in our study) overlap with the NAWQA South-Central Texas study unit (Bush et al., 2000). Groundwater is the predominant water supply for this area, and highlighted results from that study include observations that groundwater from the Trinity aquifer is generally high (greater than 500 mg/L) in dissolved solids primarily because of natural processes of water–rock interaction.

#### Columbia-Snake Region (14)

The Upper Snake Basin, which is mostly within southeastern Idaho and northwestern Wyoming, is top ranked in the region. This basin’s score stands out (Fig. 6) because of relatively high values for modeled stream conductivity and water stress (Table S2). Groundwater from wells in this basin contained some of the greatest dissolved solids concentrations of those reported in all NAWQA Study Units from across the US, and these elevated concentrations are attributed to leaching of evaporative salts and soil minerals during infiltration of irrigation water (Clark et al., 1998). The remaining basins show a relatively narrow range of scores showing no significant linear correlations to the variables or variable categories (Table 4).

#### Central Rockies Region (15)

Long-term decreasing trends in total dissolved solids in surface water have been observed in regions of the western US that are included in the Central Rockies Region and the California-Nevada Region, and these changes have been predominantly attributed to water diversions, changes in irrigation, and changes in land use (Anning & Flynn, 2015; Kaushal et al., 2018; Rumsey et al., 2021). The Upper Colorado-Dolores Basin, which straddles the Colorado-Utah border and includes Paradox Valley and its thick near-surface salt deposits, is top ranked in this region. The high score (Fig. 6) is driven by relatively high values in *Depth to Brackish* and *Conductivity* (Table S1 and S2). The second ranked Lower Green Basin similarly has relatively high predicted stream conductivity, but also a relatively high value for *Road Salt*. Otherwise, scores are relatively close to one another in this region, with *Irrigation* and *Water Stress* showing the significant correlations with overall score (Table 4). The Colorado-Gunnison Basin, the fifth ranked basin in this region, is geographically identical to the Upper Colorado River Basin selected as the second USGS Integrated Water Science basin, and also the NAWQA Upper Colorado River Basin study unit (Spahr et al., 2000), and has been the subject of much USGS research on salinity (Nauman et al., 2019; Rumsey et al., 2021).

#### Southwest Desert Region (16)

The Upper Pecos Basin, which is in eastern New Mexico, is top scoring in the region, with relatively high ranks for *Carbonate Evaporites*, *Depth to Brackish*, and *Conductivity* (Table S1 and S2). This basin is known for concerns of elevated salinity in the Pecos Rover (Houston et al., 2019). The second highest scoring basin in the region is the adjacent Rio Grande Closed Basins, which has similarly high ranks and relatively high ranks for *Water Stress* and *SocioEconomic* variable (Table S2). Across the region, overall scores are significantly correlated with *Depth to Brackish*, *Conductivity*, *Precipitation Change*, *EcoHealth*, and *SocioEconomic* rankings (Table 4).

#### Pacific Northwest Region (17)

Specific conductance and base cation concentrations in streams and rivers tend to be decreasing in the Northwest (Kaushal et al., 2018). *Rain Cl* is a relatively high Source in this region (Fig. 3), and *Precipitation Change* is a highly ranked Driver (Fig. 4). The Oregon-Washington Coastal Basin is top ranked in the region. There are only four basins in this region, and the top three ranked basins have relatively close scores (Fig. 6). A relatively high rank for the *SocioEconomic* variable puts Oregon-Washington Coastal Basin at the top (Table S2). A relatively low Driver category subscore, representing less anticipated change in precipitation or aridity, gives the Klamath-Northern California Coastal Basin a fourth rank score markedly lower than the top three (Table S2). The Willamette Basin in this study is identical to the NAWQA Willamette Basin study unit, which was remarkable in its study results that dissolved solids concentrations in groundwater were low compared to other NAWQA study units (Wentz et al., 1998).

#### California-Nevada Region (18)

The Escalante Desert-Sevier Lake Basin, which is an arid region in southwestern Utah, is top ranked in the region. This basin stands out (Fig. 6) because of its relatively high *Depth to Brackish*, *Conductivity*, *Aridification* index, and *SocioEconomic* ranks (Table S2). Overall scores in this region are best correlated with the *Aridification* and *Irrigation* ranks (Table 4). Excluding *SocioEconomic* from the ranking drives the second-ranked Great Salt Lake Basin, which lies just north of Escalante Desert-Sevier Lake Basin, into a standout top score position. That basin includes Great Salt Lake, which has been shrinking and increasing in salinity because of drought and water diversion, threatening a lake ecosystem collapse (Kintisch, 2022).

#### National scale map

For reference, a national scale map is presented as Figure S2. The ranking process is similar to the regional analysis, save for that each Basin’s score is ranked across the entire nation rather than only within the hydrologic landscape region that the basin belongs to. The highest ranked basins are generally around the Great Lakes and in the Midwest region, with the Florida peninsula, South Carolina, and Louisiana Gulf Coast also standing out. The Alabama Basin (Basin ID 0315) has a low rank that contrasts sharply with its neighboring basins, as does the Lower Cimarron-Keystone (Basin ID 1105) in the Southern High Plains Region. Both the Alabama and Lower Cimarron-Keystone basins are lower-end outliers within their respective regions. The use of regions provides an underlying framework of areas that are homogeneous in terms of the major drivers of the hydrologic cycle precipitation, temperature, and elevation and relatively unique from other areas (Van Metre et al., 2020). Ranking of basins within those regions for scientific monitoring and assessment then should give an understanding of the salinity and salinization across the US rather than in just one area. The distribution of scores is also consistent with the idea that observed changes in chloride—i.e., salinity—are not responding to a widespread, common driver across US lakes and streams (Sprague et al., 2019). The obvious contrast between low priority of arid regions and high priority of wet regions seems like it might be counterintuitive from some perspectives, and it would be undesirable to avoid doing more salinity research in major portions of the western US. In summary, the ranking system in this study is designed to work better on a regional basis.

## Discussion

There are many ways to approach ranking. Our goal was to have a well-defined approach to ranking basins in each region that considered and included many of the factors involved in salinity and changes in salinity in the light of water availability and water security. We tried to balance a simple approach with the complex ways that the geochemistry of natural waters is regulated. Our goal was not to predict water quality, but rather to highlight areas where studies of water quality issues related to salinity might be the most useful to improve understanding and prioritize science. However, this ranking approach does not capture sea level rise or other coastal salinization issues very well. Issues such as time lag in watershed response (Ilampooranan et al., 2019) are not considered in the results. The economic value of agricultural loss is not captured, which has been highlighted in the past in the Colorado River Basin when discussing the importance of salinization (Vengosh, 2014).

The use of percent ranks rather than variable values makes the relative positions of the basins in each variable more important than the actual variable values. Thus, when using variable values, some basins with major issues might not be clearly distinguished from others that do not share those issues, and many basins with similar variable values are spread out over a range of importance. In contrast, the use of percent rank to calculate scores tends to minimize the influence of outliers. Additionally, basins with major issues may already be targets of detailed investigations. We used computational methods and many data sets used in Van Metre et al. (2020) for a continuity of approach. Van Metre et al. (2020) recognized that there is no “best” approach, that variable choice and how to manipulate those variables is subjective, and that there is no objective test for these rankings. Success, they reasoned, can only be measured in carrying out the studies. Regardless, Van Metre et al. (2020) felt that their results compared favorably to a similar effort carried out by US Environmental Protection Agency (EPA) to come up with data-based indices (IWI and ICI) for watershed and catchment integrity (Thornbrugh et al., 2018) and concluded that their rankings were achieving their objective of identifying basins with high levels of anthropogenic stress on their water resources. Johnson et al. (2019) revised the IWI and ICI noting that previous values are probably over-estimates of the actual integrity of the Nation’s watersheds and catchments but concluded that the indices could be further improved as more and better data became available.

The inclusion of a variable for socioeconomic factors is a key difference in this ranking process from the previous effort by Van Metre et al. (2020). Although later steps may be included in a selection process for prioritizing salinity research—such as stakeholder input from other USGS entities, federal agencies, state and local management and nongovernmental organizations—we elected to include socioeconomic factors up front because scientists have an obligation to consider both the ability and consequences of people interacting with the environment. The *SocioEconomic* variable appeared to be an important contributing variable especially in Northern High Plains and Southwest Desert where the variable was significantly correlated with the final score (Table 4). In addition, a relatively high ranking for this variable (i.e., low mean median household income in the basin) was associated with top ranked scores in the Atlantic Coast, Southwest Desert, Pacific Northwest, and California-Nevada regions.

### Comparison with results from previous ranking and selection by USGS

The rankings developed by Van Metre et al. (2020) were based on anthropogenic stressors of water resources and ecology, with the goal to prioritize basins facing water resource challenges. In the present study, we attempt to add water quality related to salinity as part of that goal. Examining where these ranking goals align provides support to the overall idea of choosing a specific basin as a priority. The rankings appear to match very well in many regions, including Atlantic Coast, Florida, Great Lakes, Mississippi Embayment, and Souris-Red-Rainy regions. For example, in the Great Lakes Region, the first, second, and third-ranked basins in Van Metre et al. (2023) are Western Lake Michigan, Eastern Lake Michigan, and Lake Huron, respectively, so that basins within the top three ranking are the same across studies. In the Souris-Red-Rainy Region, the Missouri-Big Sioux Basin is top ranked in both this study and Van Metre et al. (2023). Other basins in that region have comparable ranks in many respects (high road salt use, high irrigation, high agricultural land use), and the scores are similar (Fig. 6) among ranks 2, 3, and 4. The Souris Basin has the lowest score and is characterized by low *Carbonate Evaporites*, *Road Salt*, and *Irrigation* (Table S2). There are only three basins in the Florida and Mississippi Embayment Regions, so a good match is not unexpected.

In other regions—the Northeast, Midwest, Northern High Plains, Southern High Plains, Texas, Columbia-Snake, and Pacific Northwest regions—the top ranks between this study and Van Metre et al., 2023 mostly overlap, but a few top ranks fall out in the comparison. For example, in the Northeast Region, Van Metre et al. (2023) identified the Delaware River Basin as their top ranked basin in this region. The Lake Erie and Ontario Basin was ranked second in Van Metre et al. (2023). In the present salinity study, the Delaware River Basin ranked 4th out of the 11 basins in the region, although the distribution of scores (Fig. 6) shows that basin still stands out from lower scores in the region. The low *SocioEconomic* rank of the Delaware River basin (0.09, Table S2) drops its overall rank substantially. Excluding *SocioEconomic* from the calculations raises the Delaware River Basin to second rank, but higher scores in the Sources category keep the Lake Erie and Ontario Basin as the top rank. In the Midwest Region, the Western Lake Erie and Upper Ohio basins (first and second in our study) rank second and sixth, respectively, in Van Metre et al. (2023). The Upper Illinois Basin, which ranks first in Van Metre et al. (2023), ranks fourth out of the fifteen basins in this region in our study. The Upper Illinois Basin has the highest *Urban* and *Road Salt* variable values in the region, but these high values are offset by a relatively high mean median income for the region that lowers the basin’s priority compared to others in our study. In the Northern High Plains Region, the Missouri-Musselshell and Missouri-Marias (first and second in our study) basins rank fifth and first, respectively, in Van Metre et al. (2023). Omitting the *SocioEconomic* variable in our ranking yields Missouri-Marias Basins as the highest ranked basin, demonstrating that it is a key difference between our approach and Van Metre et al. (2023).

In some regions—Tennessee-Missouri, Gulf Coast, Central High Plains, Central Rockies, Southwest Desert, California-Nevada—the ranks between this study and Van Metre et al. (2023) do not match well. Some of these regions, such as Southwest Desert and California-Nevada, are distinct in that they are areas where *Conductivity* and *Depth to Brackish* variables are relatively high and have high values for the top ranked basins, and therefore, differences between the present study and Van Metre et al. (2023) can be attributed to some extent to including sources of salinity. In the Gulf Coast and California-Nevada regions, the rate of change in groundwater storage variable appears to play a key role in differences. In the Gulf Coast Region, the Louisiana Coastal and Lower Mississippi Basin (first and second in our study) rank fourth and third, respectively, in Van Metre et al. (2023). That study gives the top ranked basin as Trinity-San Jacinto, which stands out predominantly because of relatively high values for the rate of change in groundwater storage variable [*GW_sto_change*] and to a lesser extent for variables *Urban* and *Ag* (see Van Metre et al. (2023) Supplemental Information Table S5). In the California-Nevada region, the top three basins in Van Metre et al. (2023) are San Joaquin, Tulare-Buena Vista Lakes, and Sacramento, in rank order, and the top ranks occur where there are relatively high values of *Tot_WU* (i.e., water stress) accompanied by relatively high *GW_sto_change* (Van Metre et al., 2023, Table S5). These top ranks differ from those in the present study, where those basins rank fourth, third, and sixth out of the thirteen basins in the region.

In some cases where there are large differences in ranking between our study and Van Metre et al. (2023), the reason appears complex. For example, in the Central Rockies Region, the Colorado-Gunnison, Great Divide-Upper Green, and Upper Arkansas are the top three basins in rank order in Van Metre et al. (2023). In the present study, those basins rank fifth, seventh, and sixth. Relatively high *Tot_WU* and *Runoff* distinguish the Colorado-Gunnison in Van Metre et al. (2023) (Table S-5, Van Metre et al., 2023). In contrast, the water stress variables (i.e., the ratio of *Tot_WU* to *Runoff*) of the Upper Colorado-Dolores and Colorado-Gunnison are similar in both studies. Comparing the Upper Colorado-Dolores and the Colorado-Gunnison basin results for this study, the higher subscore in the Drivers category of the Colorado-Gunnison did not outweigh the higher subscore in Receptors, which was driven mostly by higher *SocioEconomic* and *EcoHealth* ranks in the Upper Colorado-Dolores Basin. We note also that the economic value of agriculture and the contribution of the Colorado-Gunnison Basin to the overall salinity of the Colorado River are far higher than those of the Upper Colorado-Dolores Basin (Tuttle & Grauch, 2009). In summary, the large differences in ranks observed in the Central Rockies Region can be attributed in part to a combination of the inclusion of *Tot_WU* and *Runoff* in Van Metre et al. (2023) and the inclusion and differences in *SocioEconomic* and *EcoHealth* variables in the present study.

As of 2024, out of the ten planned USGS Integrated Water Science (IWS) basins, five have been selected: Delaware River, Upper Colorado River, Upper Illinois, Willamette, and Trinity-San Jacinto River. Each of these basins was the top ranked in their region in Van Metre et al. (2023). The Delaware River Basin was selected by USGS as a pilot location for intensive monitoring (Eberts et al., 2019) and became the first of the IWS basins. In the present study, this basin ranks 4th out of the 11 basins in the region, although the distribution of scores (Fig. 6) shows that basin still stands out from lower scores in the region. The Colorado-Gunnison is geographically identical to the Upper Colorado River Basin and was selected as the second IWS basin. In our ranking exercise, the Upper Colorado-Dolores was top ranked, and Colorado-Gunnison was fifth. As described above, the difference in results of the two approaches for the Central Rockies Region is driven to a large degree by the selection of variables in the Receptors category. The Upper Illinois Basin in the Midwest Region, which was selected as the third IWS basin, ranks fourth out of the fifteen basins in this region in the present study. The difference in Upper Illinois Basin and our selected top basin is primarily because of relative differences in *EcoHealth* and *SocioEconomic* variables. In the Pacific Northwest Region, the Willamette Basin, which ranks third in the present study, was selected as the fourth IWS Basin in 2022, and its top rank in Van Metre et al. (2023) was driven primarily by variables *Ag*, *Tot_WU*, and *Eco_sensitivity*. However, the top three ranked basins have relatively close scores (Fig. 6). The Trinity-San Jacinto River Basin in the Gulf Coast Region was selected as the fifth IWS basin in 2023. The basin stands out among others in the region in Van Metre et al. (2023) predominantly because of relatively high values for the rate of change in groundwater storage variable [*GW_sto_change*] in that study and to a lesser extent for variables *Urban* and *Ag*.

### Research needs

Research needs for the management of salinity, salinization, and water resources have been presented both outside (Cañedo-Argüelles et al., 2016; Cunillera-Montcusí et al., 2022; Kaushal et al., 2021) and within the USGS (Anning & Flynn, 2014; Evenson et al., 2013; Stanton et al., 2017; Stets et al., 2020). Key research activities for USGS IWS basins and elsewhere could include determining sources, pathways, and loadings; predicting and understanding changes; understanding the components of salinity and mobilization of contaminants; understanding the relationship between salinization and changing ecosystem structure; and furthering knowledge on the causes and distribution of groundwater salinity, brackish water resources, and challenges related to desalination (Table 5). Allowing that there is overlap across these research activities, each of these is described separately in more detail below.

**Table 5:** Summary of salinity and salinization research needs and potential directions for basins

Develop ability to distinguish various salinity sources and multiple transport pathways from sources to receptors including watershed or aquifer mass balance approaches combined with flow modeling to better understand the interactions between surface water, the unsaturated zone, and groundwater
Address understanding of hourly, seasonal, and long-term changes in sources, peaks, and trends of salinization including monitoring salinity with high-frequency sensors, developing long-term continuous data
To understand the importance of salt ions and mixtures, include a survey of data on major ion ratios and trace elements related to salinity in fresh and brackish water systems, and develop predictive models for groundwater quality based on local geologic, hydrologic, and climatic conditions
Develop water quality observation networks and research to understand how salinity affects coastal carbon sequestration, invasive species, and loss of plant communities anchoring coastal wetlands that transform nutrients and stabilize coastline
Improve knowledge in groundwater salinity, brackish water resources, and desalination including the distribution of brackish groundwater resources, the sustainable use and/or treatment of brackish groundwater resources, and water treatment and desalination technologies

#### Determining sources, pathways, and loadings

A key gap in salinity research is often the ability to distinguish various salinity sources and transport pathways. The ability to do so is crucial for management, model prediction, and remediation. Moreover, the regulations and best management practices used to address nutrient and sediment impairment may not apply to salinity and salinization issues because the sources and pathways may be substantially different (Kaushal et al., 2022; Stets et al., 2020). The determination of various sources and their relative contributions and relationship with watershed characteristics often overlaps with the examination of long-term trends (Baker et al., 2019; Rumsey et al., 2021, 2023), which is discussed in the next section.

Proposed research activities to address these gaps include watershed or aquifer mass balance approaches, which can be combined with either geochemical and isotopic tools for identifying sources or can be combined with flow modeling to better understand the interactions between surface water, the unsaturated zone, and groundwater. For example, Tuttle and Grauch (2009) used sulfur and oxygen isotopes of sulfate to fingerprint geological sources of salinity to the Upper Colorado River and major tributaries and calculated relative contributions of these sources. Mass balance approaches are more typically applied on lake or reservoir scales, such as an examination of Lake Powell as a sink for salinity in the Colorado River Basin (Deemer et al., 2020). Geochemical methods for sources of saline water are frequently applied in studies of groundwater, oilfield water, or thermal water (Kharaka & Hanor, 2014). However, tracers such as chloride to bromide ratios have been used to distinguish natural and anthropogenic sources of salinity in streams, such as road salt (Gutchess et al., 2016). An examination of trends in alkalinity—which is closely related to salinity—and multiple solutes in rivers can also provide insight into sources and drivers of salinity (Stets et al., 2014).

Combination of concentrations and loads with flow modeling can also provide insight into salinity sources. Rumsey et al. (2023) performed an analysis of stream flow and stream conductivity at 35 sites in the Delaware River basin using a Weighted Regressions on Time, Discharge, and Season (WRTDS) model and concluded that there was evidence for the increasing role of subsurface in delivering legacy salt to streams in the Delaware River Basin, consistent with observations that the unsaturated zone can be a long-term reservoir of chloride (Lax & Peterson, 2009). Quantification of land uses—urban, agricultural, impervious surfaces, crop type—using remote sensing or GIS techniques could also provide improved information on sources and pathways (Baker et al., 2019; Cunillera-Montcusí et al., 2022; Rossi et al., 2022, 2023), including an analysis on hydrologic connectivity of streams. Improving ways of understanding the relationship between groundwater flow systems and salinization is an important avenue of research, especially in coastal zones (Evans et al., 2020) and groundwater fed lake systems (Zamora & Inkenbrandt, 2024).

#### Predicting and understanding changes

Another key gap in salinity research is an understanding of hourly, seasonal, and long-term changes in sources, peaks, and trends of salinization. The importance of this knowledge is in the ability to extrapolate salinity measurements and models over a wide range of hydrologic and climate variables and gradients and time scales, including direct and indirect human impacts.

Proposed approaches to address this gap include monitoring salinity with high-frequency sensors and developing long-term continuous data. Such studies could be particularly useful in areas where human activities cause redistribution of salts or saline water or in areas where large pulses of salt are added to the environment (e.g., deicing during winter storms, Brown et al., 2015; Moore et al., 2020). For example, Shattuck et al. (2023) combined high-frequency specific conductance sensors with long-term stream and groundwater quality data to examine how long-term (multidecadal) changes are related to episodic events in 13 streams across New Hampshire, USA, with results showing that variation in stream flow, extreme events, and application of deicing agents play a role in freshwater salinization.

A focus on long-term data sets and trend analysis is another important approach to understanding change. For example, in the Upper Colorado River Basin, a study using WRTDS model showed substantial decreases in dissolved-solids loads and concentrations in streams from 1929 to 2019 (Rumsey et al., 2021). WRTDS modeling was also applied to show widespread increases in specific conductivity in the Delaware River Basin (Rumsey et al., 2023) and watersheds in southeastern Pennsylvania (Rossi et al., 2022, 2023). Baker et al. (2019) combined a 25-year time series of impervious cover from remote sensing and watershed monitoring for stream conductivity in the Baltimore-Washington, DC, metropolitan area to find increases in conductivity from complex contributions of urbanization, road salt application, and other factors. Both these studies highlight the importance of collecting and using previous long-term datasets, combining with land use data, and examination using advanced statistical and modeling techniques. Both the studies also overlap with an examination of sources, pathways, and loading of salinity. A recent ecological-focused gap analysis of salinization highlighted the rarity of long-term studies and the critical importance of doing more (Cunillera-Montcusí et al., 2022). In an examination of different sampling approaches to detect changes in chloride concentrations in US rivers and streams, Sprague et al. (2019) showed different results for probabilistic and targeted methods and thus concluded that there is a need for robust monitoring combining those methods to capture trends.

Paleolimnological data offer another approach to examining long-term datasets related to salinity. For example, Cladocera fossil assemblages in lake sediment cores were used to investigate effects of road-salt application in lakes in Southern Ontario, Canada (Valleau et al., 2020). Diatom assemblages have been used to assess salinity (Frost et al., 2023), but not widely applied in the US although metrics developed using diatom assemblages have been associated with water quality indicators including salinity (Carlisle et al., 2022). Development of harmonized datasets may provide an opportunity to further investigate changes in water chemistry (Potapova et al., 2022).

#### The components of salinity and mobilization of contaminants

There is uncertainty regarding how different salt ions and mixtures affect biota, ecological communities, and ecosystem functions and services, as well as uncertainty on how these different saline waters affect beneficial uses, mobilization of contaminants, and compatibility of water with water treatment technologies (Kaushal et al., 2021, 2022; Stets et al., 2020). The importance of understanding this relates to the mobilization of toxic chemicals and nutrients that affect biodiversity and ecosystem processes. The importance of mixtures is also evident in how different major ion ratios have different properties related to corrosion, scaling, or water treatment processes that may affect drinking water infrastructure. Ion ratios in irrigation water can affect soil fertility, decrease infiltration, or have undesirable effects on the chemistry of agricultural return water (Stanton et al., 2017; Vengosh, 2014).

Proposed approaches to understanding the importance of these mixtures and “chemical cocktails” include a survey of data on major ion ratios and trace elements related to salinity in fresh and brackish water systems and the development of predictive models for geochemical water type in groundwater based on local geologic, hydrologic, and climatic conditions. For example, Stanton et al. (2017) used cluster analysis to classify saline groundwater in the US into four distinct groups based on major cations, major anions, silica, dissolved-solids concentration, pH, and temperature, noting that there were notable spatial patterns in the regional distribution on a scale of tens to hundreds of miles. Further examination of these regional patterns could lead to a better understanding of natural sources of potentially toxic elements in groundwater and provide a basis to extrapolate to other areas with less or no data. Processes resulting from salinity changes such as mixing, ion exchange, and mineral dissolution or precipitation must be assessed using geochemical or reaction transport models and incorporated into other water-quality models (Lombard et al., 2020). This approach is especially important where sea level rise and anthropogenic inputs such as road salt are altering groundwater chemistry (Lindsey et al., 2021; Vinson et al., 2013). Some of these approaches overlap with those discussed under the section on groundwater salinity and brackish water resources below.

#### Changing ecosystems

Understanding of effects of salinization (e.g., sea level rise and saltwater intrusion) on ecosystem health and community structure is another critical gap. Salinity changes affect coastal carbon sequestration, habitat ranges of invasive species, and loss of plant communities anchoring coastal wetlands that transform nutrients and stabilize coastline. In a review of ecological gaps in freshwater salinization, Cunillera-Montcusí et al. (2022) also highlighted that specific habitats such as small ponds may be under-represented in research despite their key role in biodiversity and providing ecosystem services and the importance of studying small changes in salinization in oligohaline systems such as high mountain streams. The impact of freshwater salinization on primary production, microbial activity, and higher trophic levels is another critical area of research. Some proposed approaches to understanding these changes include using detailed (high-frequency sensor) investigation of salinity controls on dissolved organic matter quantity and composition in coastal water and on microbial products. Monitoring environmental DNA which can be used to detect invasive species and assess biodiversity could also be combined with salinity monitoring, although techniques may need to be developed for specific freshwater or estuarine habitats or applications (Harper et al., 2019; Nagarajan et al., 2022; Sepulveda et al., 2020). In coastal regions, the Coastal Salinity Index (CSI) is a long-term monitoring tool that could be applied to understanding the effects of changing salinity conditions on fresh and saltwater ecosystems, fish habitat, and freshwater availability for municipal and industrial use (Petkewich et al., 2019).

#### Groundwater salinity, brackish water resources, and desalination

Across the US, the demand for water is increasing as water quality is degrading, and some regions are becoming more arid. The importance of brackish and saline water in meeting future water resource challenges is recognized within the US and worldwide (Evenson et al., 2013; Vengosh, 2014). Consequently, there are important opportunities for improved knowledge of the availability and sustainability of groundwater and saline water resources (Evenson et al., 2013; Kaushal et al., 2021; Stanton et al., 2017). Accordingly, the USGS has proposed specific work to develop and apply methods to quantify the potential for expanded future use of these and other alternative water sources (Evenson et al., 2013; Stanton et al., 2017). Some proposed approaches to addressing these knowledge gaps include estimating groundwater salinity in three dimensions across the contiguous US where chemical data are not available (using methods similar to efforts on nitrate in groundwater by Ransom et al., 2022), characterizing the distribution of brackish groundwater resources; refining knowledge of the sustainable use and/or treatment of brackish groundwater resources; and conducting research related to water treatment and desalination technologies and limitations. For example, because of the potential importance of increasing saline water use in the future, the distribution of saline groundwater and the baseline chemistry of aquifers must be characterized where feasible (Stanton et al., 2017). As part of this baseline understanding, links between source-water geochemistry, required water treatment, and potential end users should be examined with the aid of geochemical modeling and simulations in different hydrogeologic settings (McMahon et al., 2016). Furthermore, data from multiple sources—local reports, oil and gas production wells, geophysical and lithologic logs, and numerical models—could be compiled to provide estimates of needed parameters for regional assessments of priority principal aquifers through the generation of maps and summaries of geochemical data (Stanton et al., 2017).

## Conclusion

In this work, we attempt to offer a science-based approach for ranking 163 basins across 18 hydrologic regions for salinity-focused research. As with the original prioritization by Van Metre et al. (2020) based on anthropogenic stress in watersheds, the ranking of basins here is a preliminary step that must also include input from USGS Water Science Centers, other USGS Mission Areas, other federal agencies, state and local environmental management agencies, and various nongovernmental organizations. Input from similar water quality parameter ranking studies—nitrogen, sediment, temperature, contaminants of emerging concern, geogenics—could also advise priorities (Erickson et al., 2024; Tesoriero et al., 2024). We note that there is no assessment of the relative importance of regions in this study, i.e., whereas salinity is a known issue in the Central Rockies and Northeast regions, the relative priority of these regions over the Columbia Snake, Pacific Northwest, or other regions is not considered.

The research activities highlighted for potential study above draw from our own synthesis of previous studies as well as previous research suggestions (Cunillera-Montcusí et al., 2022; Kaushal et al., 2021; Stanton et al., 2017) with a particular focus on work within the scope, strengths, and previous work of the USGS Water Mission Area, which includes its streamgaging monitoring network, water quality measurements, and long-term datasets. These research ideas presented are not meant to be comprehensive for the research community, and USGS resources and priorities can change. Moreover, additional issues or new ways of approaching problems can arise. For example, artificial intelligence forms such as machine learning and big data have the potential to transform how data are collected and analyzed (Beibei et al., 2023; Knierim et al., 2020). Nevertheless, the prioritization, gap analysis, and potential approaches presented above are intended to aid the USGS community and beyond in addressing the growing challenge of the salinization of freshwater and groundwater systems.

## Supplementary Information

Below is the link to the electronic supplementary material.

Supplementary file1 (DOCX 5684 KB)

Supplementary file2 (XLSX 104 KB)

## Data Availability

A detailed description of data sources and how data were prepared and processed to the basin scale is provided in a USGS Data Release by Qi et al. (2023). Qi et al. (2023). Geospatially derived environmental characteristics to prioritize watersheds for research and monitoring needs within 18 hydrologic regions across the United States. U.S. Geological Survey data release. https://doi.org/10.5066/P9KFU815.
